# Impact of zinc oxide, benzoic acid and probiotics on the performance and cecal microbiota of piglets

**DOI:** 10.1186/s42523-021-00151-y

**Published:** 2021-12-20

**Authors:** Caio Abercio da Silva, Leonardo Aparecido Teixeira Bentin, Cleandro Pazinato Dias, Marco Aurélio Callegari, Vitor Barbosa Facina, Francine Taniguchi Falleiros Dias, Adsos Passos, Cláudia Cassimira da Silva Martins, Marcio Carvalho Costa

**Affiliations:** 1 Department of Animal Science, Universidade Estadual de Londrina, Londrina, Paraná Brazil; 2 Department of Clinics, Surgery and Animal Reproduction, São Paulo State University (Unesp), Araçatuba, São Paulo Brazil; 3 Department of Veterinary Biomedical Sciences, Université de Montréal, Saint-Hyacinthe, Québec Canada; 4 Akei Animal Research, Fartura, São Paulo Brazil; 5 DSM Nutritional Products Ltd., Jaguaré, São Paulo Brazil

**Keywords:** Antibiotics, Diarrhea, Organic acids, Swine

## Abstract

**Background:**

Intestinal health remains a key factor in animal production because it is essential for digestion, absorption and bacterial fermentation. Feed additives have been used to attenuate the weaning stress such as Zinc Oxide (ZnO) and benzoic acid (C_7_H_6_O_2_). The objective of this study was to evaluate the impact of of benzoic acid and probiotics (BA + P) on performance, diarrhea and cecal microbiota of piglets in the nursery phase (23 to 65 days).

**Results:**

One hundred and sixty weaned piglets with an initial weight of 6.335 ± 0.698 kg and 23 days of age were submitted to four treatments: supplementation with 2500 ppm of Zinc oxide (ZnO), supplementation with a commercial blend of benzoic acid and probiotics (*Bacillus licheniformis, Bacillus subtilis* and *Enterococcus faecium* NCIMB 10415; Vevogut P®) (BA + P), supplementation with Zinc oxide plus benzoic acid and probiotics (ZnO + BA + P), and controls receiving only the basal diet without any supplementation. At 65 days of age, 32 piglets (n = 8 per treatment) were slaughtered for the evaluation of the cecal microbiota. Supplementation with ZnO and BA + P were associated with better feed conversion (*P* < 0.05) in the early stage (23 to 49 days) and with an improvement in all performance parameters over the entire experimental period. The occurrence of diarrhea was lower (*P* < 0.05) in the BA + P group. The 4 most abundant phyla along with unclassified bacteria represented 93% of all sequences. Firmicutes dominated the cecal microbiota of all groups, followed by Bacteroidetes. Richness represented by the observed number of genera and by the Chao index were statistically lower in ZnO and ZnO + BA + P supplemented animals compared to controls. The beta diversity analysis that compares similarities between bacterial communities demonstrated formation of two distinct clusters containing samples with and without supplementation with ZnO, confirming a strong influence of ZnO on the intestinal microbiota.

**Conclusion:**

The use of Benzoic acid with probiotics yields similar performance results with lower impact on the gut microbiota compared to ZnO, and it should be considered as a potential alternative in swine production.

**Supplementary Information:**

The online version contains supplementary material available at 10.1186/s42523-021-00151-y.

## Backgound

Intestinal health remains a key factor in animal production because it is essential for adequate food digestion, nutrients absorption and energy production by bacterial fermentation. Furthermore, it is the place of action where pathogens cause concerning diseases in swine production. During the first weeks of life, the gastro intestinal tract passes through several adaptation processes that may have an impact on animal performance [1–3] Weaning is the most critical phase in swine production and post-weaning diarrhea remains the most common cause of morbidity and mortality in swine herds resulting in great economical losses [4–6].

Several strategies to attenuate the negative impact of the stress observed during weaning have been evaluated [7, 8]. Feed additives, such as Zinc Oxide (ZnO) [9] and benzoic acid (C_7_H_6_O_2_) [7] are globally used in swine production systems. The use of those products is associated with lower incidence of diarrhea and greater weight gain during weaning [10, 11]. Zinc is essential for the intestinal mucosa integrity and protects against pathogenic organisms by modulation of the immune system [12], but the excessive use of ZnO during the last decade as an alternative for antibiotic growth promoters has been associated with the development of bacterial resistance to this product [13] and with environmental pollution [14]. Therefore, alternatives to this product such as essential oils and probiotics deserve further investigation.

The importance of the intestinal microbiota, especially of bacteria, has been revised after the development of next generation DNA sequencing (NGS) technologies, which are much more comprehensive than traditional culture-based approaches. Several studies have used NGS to reveal that the swine intestinal tract harbours a complex bacterial community that can vary according to age, diet and management [15]. The intestinal microbiota has been shown to be essential for the maintenance of intestinal health and to play a major role in the development of the local and systemic immune system. Therefore, microbiota manipulation has been attempted with the objective of improving health [16]. Amongst the different strategies to modulate the intestinal microbiota, the use of probiotics is a viable option to be used in swine production [17–19].

Dietary supplementation with ZnO affects the intestinal microbiota of pigs [13, 20], but studies comparing the impact of different products in a controlled environment remain to be performed. This study aimed to investigate the impact of ZnO, benzoic acid and probiotics on growth performance, control of diarrhea and cecal microbiota composition of piglets at the nursery phase.

## Material and methods

### Study design

One hundred and sixty weaned piglets (Camborough PIC x AG 337 sires) at 21 days of age were raised in an experimental unit (Akei Animal Research, Fartura, São Paulo, Brazil), according to a random design of four experimental groups: supplementation with 2500 ppm of Zinc Oxide (ZnO), supplementation with 0.4% of a commercial blend of benzoic acid and probiotics (*Bacillus licheniformis, Bacillus subtilis* and *Enterococcus faecium* NCIMB 10415; Vevogut P®, DSM Animal Nutrition) (BA + P), supplementation with Zinc oxide plus benzoic acid and probiotic (ZnO + BA + P), and controls receiving only the basal diet without supplementation.

Piglets were housed in 2,55 m^2^ pens (2 females or 2 males per pen) with controlled temperature. Therefore, each experimental group contained 40 animals (20 males and 20 females) housed in 10 different pens.

Additional file 1: Tables S1 and S2 specify the composition of diet used at the different growing phases: Pre starter I (21 to 28 days), pre-starter II (29 to 35 days), starter I (35 to 49 days) and starter II (49 to 63 days). Basal diet consisted of corn and soybean meal, with water and mashed ration offered ad libitum.

### Performance

Piglets were weighed individually on day 0 (23 days of age), 5 (28 days of age), 12 (35 days of age), 26 (49 days of age) and 40 (63 days of age) of the trial to calculate average daily gain (ADG) for each period and considering all phases. Daily feed intake was determined by pen, and mortality was recorded to adjust the feed conversion ratio.

Fecal consistency was evaluated daily and classified as normal (score 0), soft (score 1), loose (score 2), or diarrhea (score 3) [21]. A diarrhea index expressed in percentage was calculated as 100 × number of piglets that had diarrhea/total number of piglets [22].

Thirty-two piglets (8 per treatment, being four barrows and four females, randomly chosen) were slaughtered at 65 days of life and cecal content was aseptically collected and immediately refrigerated for transportation and frozen at − 80 °C until DNA extraction. DNA was extracted using a commercial kit DNeasy PowerSoil (QIAGEN, Hilden, Germany) and the V4 region of the 16S rRNA gene amplified using the primers 515F: GTGCCAGCMGCCGCGGTAA and 806R: GGACTACHVGGGTWTCTAAT. Amplicons were then sequenced with a MiSeq Illumina sequencer (Illumina, Inc., San Diego, CA, USA) single end sequencing using a V3 kit for 300 bp reaction.

Bioinformatic analysis was carried out using the software mothur [23], following the protocol suggested by Kozich et al. (2013) [24]. Reads containing more than 300 bp or more than 8 homopolimers were excluded. Good quality reads were aligned to the SILVA reference alignment and classified according to the Ribosomal Database Project [25], 2016 release. Chimeras were removed with the vsearch algorithm. Reads classified as Chloroplast, mitochondria, Archaea or eukaryota were removed from the analysis. The phylotype approach was used by grouping all reads belonging to the same genus (94% similarity). Subsampling using the smallest number of reads obtained in a sample was used to standardize non-uniform samples in an attempt to avoid introducing bias into the analysis. The software mothur was also used to calculate richness by the total number of observed genera and by the Chao index. Diversity was estimated by the Simpson’s index. Community membership, which takes into account each genus present in a community, was calculated by the Jaccard index, and community structure, which takes into account each genus and their relative abundances, was calculated by the Yue and Clayton index. The distance matrices created for both, membership and structure were subsequently used to generate dendrograms and Principal Coordinate Analysis (PCoA) graphs for visualisation of similarity between samples.

### Statistical analysis

Body weight, ADG, DFI and FRC were submitted to analysis of variance (ANOVA) and means were compared by Tukey’s test. The mean of the diarrhea index was compared between groups by the chi-squared test. Richness and diversity indices were compared between groups using an ANOVA test with Bonferroni correction for multiple comparisons using GraphPad Prism 7.0a (GraphPad Software Inc., California, USA), considering *P* values < 0.05 as significant. Relative abundances were compared by Tukey’s test.

Beta diversity (Jaccard and Yue and Clayton indices) were compared using the analysis of molecular variance (AMOVA) test. Linear Discriminant Analysis Effective Size (LEfSe) was used to find bacterial taxa significantly associated to each treatment [26].

## Results

### Performance

Animals supplemented with Benzoic acid plus probiotic and ZnO + Benzoic acid plus probiotic had significantly better FCR than the control group during the Pre-Starter I phase. Those groups, along with ZnO supplemented animals also performed better compared to controls during the Starter I phase I (Table 1). Final weight at Starter II was significantly higher in Benzoic acid plus probiotic likely because they started this phase heavier due to better performance at Starter I, as there was no statistical difference in ADG (despite a numerically greater average in that group). Overall, ZnO and Benzoic acid plus probiotic treatments significantly improved daily feed intake (DFI) and average daily gain (ADG) compared to controls.

**Table 1:** Mean values of live weight, daily feed intake (DFI), average daily gain (ADG) and feed conversion rate (FCR) of weaning piglets supplemented with Zinc Oxide (ZnO), Benzoic acid plus probiotic (BA + P) and ZnO + BA + P according the phases

Phases	Treatment				CV %	*p*-value
	Control	ZnO	Benzoic acid + Probiotic	ZnO + Benzoic acid + Probiotic		
*Pre starter I (23–28d)*						
Initial weight, kg	6.343	6.331	6.330	6.357	1.20	0.835
DFI, kg	0.212	0.223	0.228	0.221	25.61	0.932
ADG, kg	0.135	0.153	0.162	0.170	28.76	0.351
FCR	1.672^b^	1.479^ab^	1.419^a^	1.418^a^	12.87	0.017
Final weight, kg	7.290	7.406	7.465	7.403	5.81	0.831
*Pre starter II (28–35d)*						
DFI, kg	0.410	0.454	0.465	0.430	13.36	0.183
ADG, kg	0.311	0.359	0.370	0.337	16.73	0.126
FCR	1.380	1.280	1.265	1.270	14.35	0.470
Final weight, kg	9.156	9.561	9.686	9.428	6.03	0.213
*Starter I (35–49d)*						
DFI, kg	0.508^b^	0.565^ab^	0.606^a^	0.550^ab^	9.99	0.004
ADG, kg	0.281^b^	0.369^a^	0.373^a^	0.367^a^	12.34	< 0.001
FCR	1.834^b^	1.540^a^	1.640^a^	1.498^a^	10.98	< 0.001
Final weight, kg	11.968^b^	13.253^a^	13.421^a^	13.100^a^	6.02	< 0.001
*Starter II (49–63d)*						
DFI, kg	0.983	1.042	1.063	1.037	7.69	0.158
ADG, kg	0.557	0.556	0.588	0.549	9.17	0.347
FCR	1.780	1.874	1.808	1.900	7.22	0.184
Final weight, kg	21.035^b^	22.150^ab^	22.841^a^	21.935^ab^	5.33	0.015
*Total (23–63d)*						
DFI, kg	0.635^b^	0.682^a^	0.704^a^	0.671^ab^	6.18	0.007
ADG, kg	0.373^b^	0.405^a^	0.423^a^	0.397^ab^	7.44	0.006
FCR	1.711	1.683	1.664	1.681	5.31	0.703

^a, b^Means with different letters correspond to significance by Tukey’s Test (*P* < 0.05)

The diarrhea ocurrence (number of piglets that had diarrhea) and the diarrhea index (100 × number of piglets that had diarrhea/total number of piglets) are presented in Table 2. Results of statistical analysis showed that controls presented higher frequency of severe diarrhea (score 3) and of intermediary and severe diarrhea together (score 2 + 3), suggesting that supplementation with ZnO and/or Benzoic acid plus probiotic was associated with lower incidence of diarrhea. No deaths were recorded during the study period.

**Table 2:** Mean values of diarrhea occurrence and index observed in post-weaning piglets supplemented with Zinc Oxide (ZnO), Benzoic acid plus probiotic (BA + P) and ZnO + BA + P

Parameters	Treatment			
	Control	ZnO	BA + P	ZnO + BA + P
*Diarrhea occurrence*				
Score 2	11^b^	1^a^	4^ab^	3^ab^
Score 3	38^c^	21^b^	18^ab^	8^a^
Score 2 + 3	49^b^	22^a^	22^a^	11^a^
*Diarrhea index, %*				
Score 2	27,5^b^	2,5^a^	10^ab^	7,5^ab^
Score 3	95^c^	52,5^b^	45^ab^	20^a^

Means with different letters means significance by qui-square Test (*P* < 0.05)

### Microbiota analysis

A total of 2,432,186 good quality sequences were retained for the final analysis, with an average of 76,006 reads per sample (SD 7048). A subsample using the smallest number of reads obtained in a sample (1310 reads) was used to standardize non-uniform samples in an attempt to avoid introducing bias into the analysis. This approach yielded 99.21% (SD, 0.15%) coverage indicating that most of the genera present in the samples were adequately detected.

**Fig. 1:**
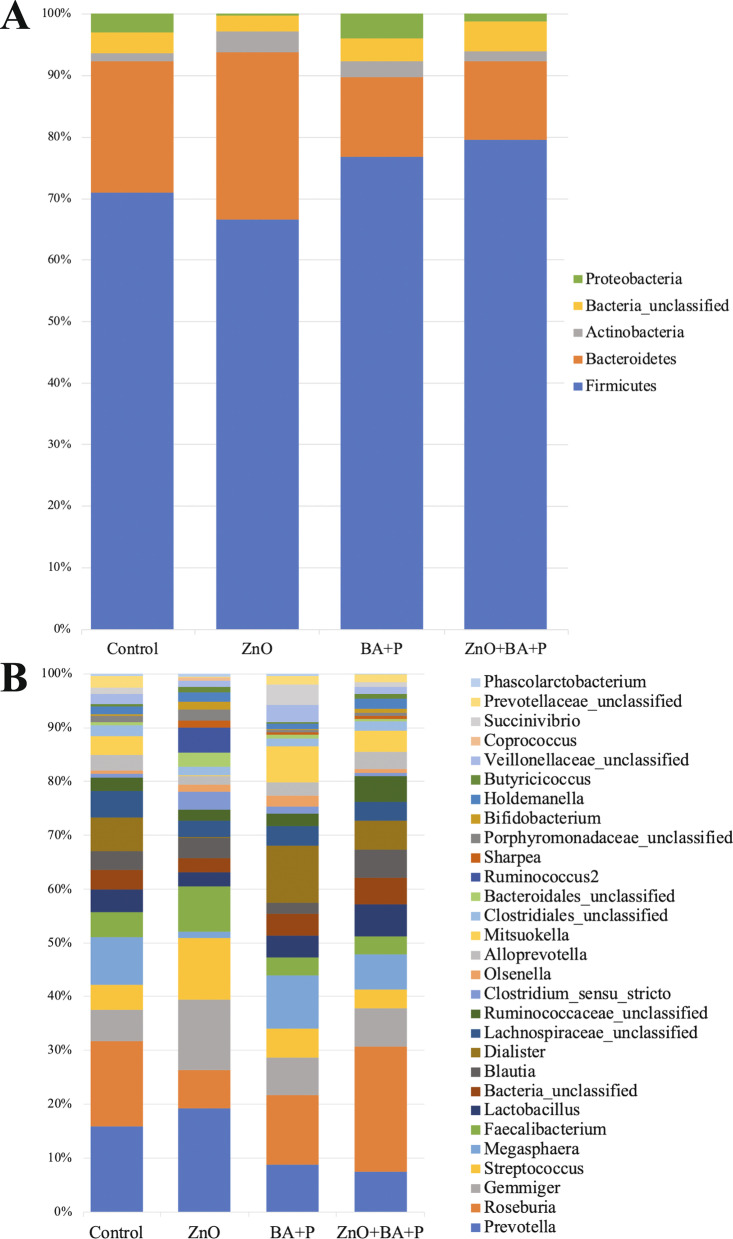
Relative abundance of the main bacteria found in the swine cecum. Relative abundance of the phyla (**A**) and genera (**B**) with > 1% of abundance present in the cecal microbiota of piglets supplemented with Zinc Oxide (ZnO), Benzoic acid plus probiotics (BA + P); ZnO + BA + P, and without supplementation (Control)

Figure 1A represents the overall relative abundance of the main phyla (> 1% abundance) found in the swine cecum. The 4 most abundant phyla along with unclassified bacteria represented 93% of all sequences. Firmicutes dominated the cecal microbiota of all groups, followed by Bacteroidetes. Figure 1B represents the relative abundances of the main genera found in the cecum of pigs supplemented with ZnO and/or Benzoic acid plus probiotic. The most abundant taxa were *Roseburia*, *Prevotella*, *Gemmiger*, and *Streptococcus* spp. Although some visual differences between treatments can be observed from Fig. 1B, there were no statistical differences found at the phylum or genus levels. The Linear Discriminant Analysis Effective Size (LefSe) analysis also failed to identify bacterial taxa associated to each of the specific treatments.

**Fig. 2:**
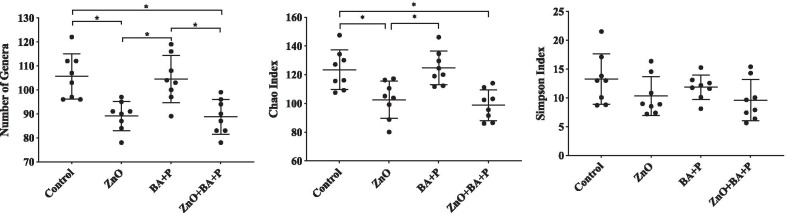
Alpha diversity indices. Number of observed OTUs (**A**), Chao index (**B**) and Simpson’s index (**C**) observed in the cecum of piglets post-weaning supplemented with Zinc Oxide (ZnO), Benzoic acid plus probiotic (BA + P), ZnO + BA + P, and no supplementation (Control). *P ≤ 0.05

Results of the statistical analysis comparing richness and diversity indices between experimental groups are shown in Table 3 and Fig. 2. Richness represented by the observed number of genera and by the Chao index were statistically lower in ZnO and ZnO + Benzoic acid plus probiotic supplemented animals compared to controls, suggesting a strong influence of ZnO on the number of different bacteria comprising the cecal microbiota. Results also show that supplementation had no influence on bacterial diversity of studied animals addressed by the Simpson’s index.

**Table 3:** *P*-values of the statistical analysis comparing alpha diversity indicators between treatments and controls

Groups	Number of Genera	Chao	Simpson
Control vs. ZnO	0.0026*	0.0132*	0.6143
Control vs. Benzoic acid + probiotic	> 0.9999	> 0.9999	> 0.9999
Control vs. ZnO + Benzoic acid + probiotic	0.0021*	0.0026*	0.2591
ZnO vs. Benzoic acid + probiotic	0.0054*	0.0077*	> 0.9999
ZnO vs. ZnO + Benzoic acid + probiotic	> 0.9999	> 0.9999	> 0.9999
Benzoic acid + probiotic vs. ZnO + Benzoic acid + probiotic	0.0043*	0.0015*	> 0.9999

^*^P ≤ 0.05

**Fig. 3:**
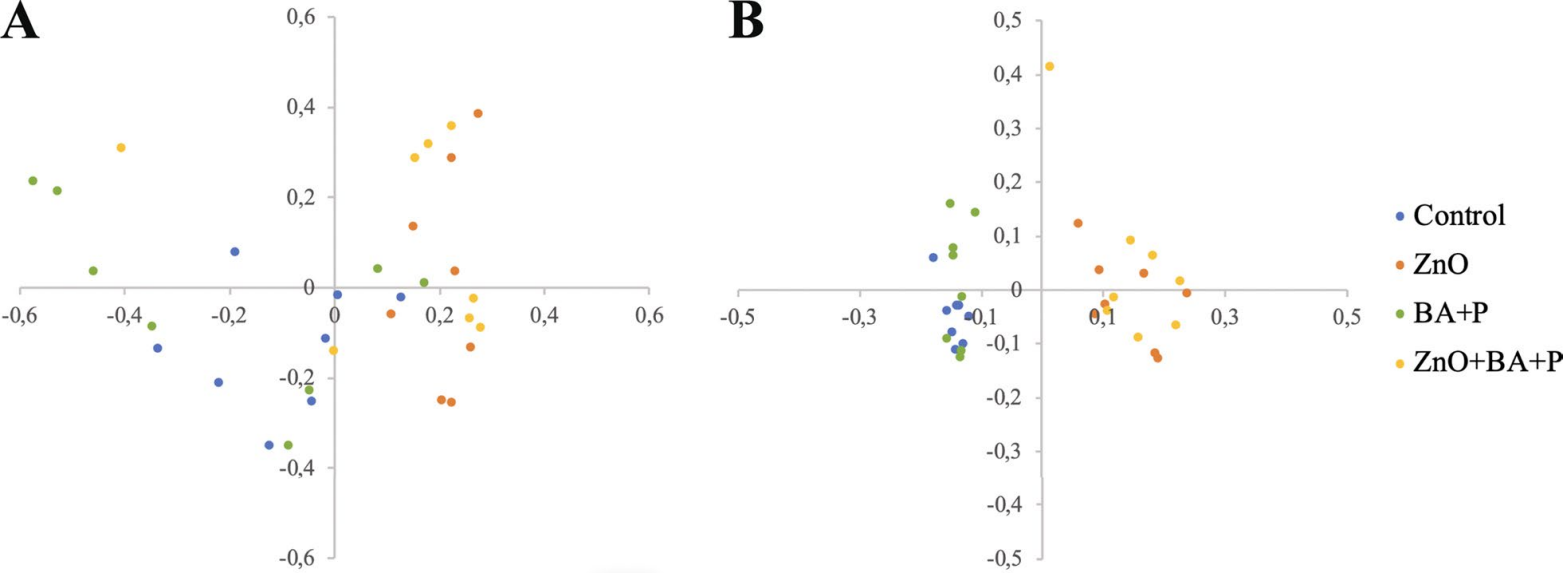
Principal coordinate analysis (PCoA). PCoA comparing the similarities between bacterial community structure (**A**) and membership (**B**) present in the cecum of post-weaning piglets supplemented with Zinc Oxide (ZnO), Benzoic acid plus probiotic (BA + P), ZnO + BA + P, and without supplementation (Control)

**Fig. 4:**
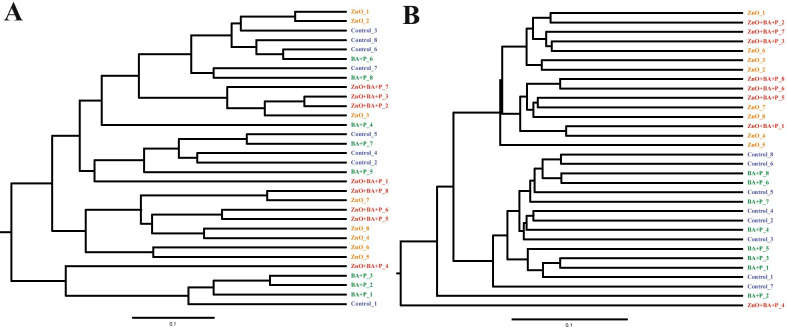
Dendrograms. Dendrograms comparing the similarities between bacterial community structure (**A**) and membership (**B**) present in the cecum of post-weaning piglets supplemented with Zinc Oxide (ZnO), Benzoic acid plus probiotic (BA + P), ZnO + BA + P, and without supplementation (Control)

The beta diversity analysis that compares similarities between bacterial communities is represented by the Principal Coordinate Analysis (PCoA) (Fig. 3). This analysis demonstrated formation of two distinct clusters containing samples with and without supplementation with ZnO. Those differences were even more evident in communities’ membership (Fig. 3B) confirming that ZnO changes the overall microbiota composition, affecting mainly the less abundant organisms. This finding was further evidenced by dendrograms that demonstrated the similarity of the microbiota membership of animals receiving ZnO (Fig. 4). The strong impact of ZnO on the cecal microbiota of pigs was confirmed by the statistical analysis (AMOVA and Parsimony) comparing beta diversity between the experimental groups (Table 4).

**Table 4:** *P*-values obtained by statistical analysis (AMOVA and Parsimony tests) comparing the similarity between bacterial communities in post-weaning piglets supplemented with ZnO and/or benzoic acid

Grupos	Structure		Membership	
	AMOVA	Parsimony	AMOVA	Parsimony
Control vs. ZnO	0.001*	0.220	0.008*	< 0.001*
Control vs. Benzoic acid + probiotic	0.452	0.965	0.975	0.388
Control vs. ZnO + Benzoic acid + probiotic	0.009*	0.199	0.005*	< 0.001*
ZnO vs. Benzoic acid + probiotic	0.003*	0.213	0.007*	0.001*
ZnO vs. ZnO + Benzoic acid + probiotic	0.452	0.787	1.000	0.270
Benzoic acid + probiotic vs. ZnO + Benzoic acid + probiotic	0.012*	0.532	0.059	0.001*

^*^P ≤ 0.05

## Discussion

As expected, animals supplemented with feed additives had better performance compared to controls, which might be related with intestinal health (lower rates and severity of diarrhea) and to the improved digestibility of nutrients [27, 28].

Zinc oxide aids to preserve intestinal mucosa integrity, modulating the immune system and protecting it against pathogenic bacteria [12]. Higher concentrations of ZnO have been associated with greater weight gain [17]. In the present study, the use of ZnO had a strong impact on the microbiota composition of the studied piglets as revealed by community analysis and reduced richness. Many studies have reported changes in intestinal bacteria of pigs caused by ZnO [29, 30]. The mechanisms by which ZnO improves growth performance are not completely understood, but it might increase the intestinal absorpiteve capacity by increasing the villus height [31].

Non-dissociated benzoic acid passes through the cellular membrane of bacteria releasing protons that acidify the medium [32], changing the bacterial metabolism and suppressing essential enzymes, such as decarboxylases and catalases [33, 34] Furthermore, benzoic acid improves the structure of the intestinal mucosa, reducing the crypt depth and increasing the villus/crypt ratio culminating in improved digestibility and performance [35–37].

Probiotic organisms (i.e. *Bacillus cereus, E. faecium, Saccharomyces cerevisae boulardii)* can improve intestinal absorption and increase the transport of L-glutamine and ion secretion in piglets, preserving the mucosa integrity by reducing enterocyte mortality and improving protein digestibility [21, 37, 38]. *Bacillus* and *Enterococcus* are among the most used probiotics because of their potential to depleat pathogenens from the digestive tract and benefit commensal bacteria, controlling for diarrhea and improving performance [21, 39, 40]. The use of a specific strain of *Ligilactobacillus salivarius* was associated with increased clostridia and lactobacilli species as well as greater production of volatile fatty acids [19]. The use of symbiotics (a combination of probiotics with prebiotics) have also been shown to benefit fibrolytic bacteria, improving performancing and reducing inflammation in pigls [18].

Firmicutes and Bacteroidetes were the two major phyla found in the cecum of the studied piglets, which was in agreement with other studies [41–44]. Although not statistically significant, the abundance of Proteobacteria was higher in the groups not receiving ZnO (Fig. 1B). The inhibition of some Proteocateria species, such as *Escherichia* and *Salmonella* may have contributed to the lower rates of diarrhea and better performance observed in the present study [45–49]. Further studies using larger sample sizes are necessary to confirm this hypothesis. Specific taxa could not be associated to each of the treatment groups, likely because of interindividual variabilities in their relative abudances. The high abundance of *Prevotella* reported here can be explained by the increase in those organisms after weaning in piglets mainly related with the fibrolytic activity of the genus [47, 50, 51]. The genus Roseburia was also highly abundant in the intestinal tract of all studied piglets. These butyrate producing bacteria have been shown to be part of the normal microbiota of piglets [52] and belongs to the family Lachnospiraceae (Firmicutes), which is normally associated good intestinal health [53]. The genus Gemmiger is another Firmicutes of the Ruminococcaceae family that was highly abundant in this study. This bacterium seems to be favoured by the insertion of soybean meal in the diet of piglets [54], which might explain our results since this protein source was an important component of the diet used in the present study. The abundance of Megasphaera was numerically lower in the cecum of piglets receiving ZnO only, but the role of these commensal bacteria in the swine intestines deserves better attention [55].

Weaning is the most stressing event in swine production due to abrupt diet changes, displacement to new environments and housing with new individuals. Not surprisingly, this is the phase with the highest incidence of diarrhea and with the highest use of antibiotics [56]. The association of benzoic acid with probiotics and prebiotics has been suggested to have a synergic effect to favor weaned piglets’ performance [37, 57, 58], which are consistent with the present study. A recent study reported that supplementation of ZnO (1200 ppm) with a probiotic containing *Bacillus coagulans, B. licheniformis, B. subtilis* and *C. butyricum* had similar effect than higher doses of ZnO (3000 ppm) on many performance paramethers [1, 17].

Although the methods employed for this study are rather descriptive and not intended to prove a cause consequence relationship, it can be hypothesized that the microbiota changes induced by ZnO may play a role in weight gain by acting at different levels, such as modulating the local immunity, benefiting species that are more efficient in extracting energy from food or by competing with pathogenic organisms. Noteworthy, the beneficial effects of benzoic acid plus probiotic observed in the present study (i.e. on growth performance and diarrhea control) were similar to the ones obtained with the use of ZnO without inducing detectable changes in microbiota composition, avoiding the negative aspects of ZnO usage, such as development of resistance and environmental contamination.

## Conclusions

The use of ZnO is associated with decreased richness and with changes in microbiota composition of weaning piglets. The use of Benzoic acid with probiotics yields similar performance results with lower impact on the gut microbiota compared to ZnO, and it should be considered as a potential alternative in swine production.

## Supplementary Information

**Additional file 1**. Ingredients and calculated composition as-fed of the experimental diets of phases Pre-starter I and II (Table S1) and Starter I and II (Table S2).

## Data Availability

Sequencing data is available at the Sequence Read Archive (SRA) – NCBI under the accession number PRJNA758076.

## Abbreviations

ZnO: Zinc oxide
BA: Benzoic acid
P: Probiotics
AGPs: Antibiotic growth promoters
NGS: Next generation sequencing
DFI: Daily feed intake
ADG: Average daily gain
FCR: Feed conversion rate
SD: Standard deviation
LEfSe: Linear discriminant analysis effective size
SRA: Sequence read archive
NCBI: National Center for Biotecnology Information
PCoA: Principal coordinate analysis
AMOVA: Analysis of molecular variance
