# Supplementary material for: Impact of zinc oxide, benzoic acid and probiotics on the performance and cecal microbiota of piglets
Source: Anim Microbiome. 2021 Dec 20;3:86. doi: 10.1186/s42523-021-00151-y (PMC8686666; doi:10.1186/s42523-021-00151-y)
Supplement: Supplementary file 1 — Additional file 1. Ingredients and calculated composition as-fed of the experimental diets of phases Pre-starter I and II (Table S1) and Starter I and II (Table S2). [file 42523_2021_151_MOESM1_ESM.docx]

**Supplementary Table 1.** Ingredients and calculated composition as-fed of the experimental diets (Pre-starter phases I and II)

| **Ingredients** | **Pre-starter I phase** | | | | **Pre-starter II phase** | | | |
| --- | --- | --- | --- | --- | --- | --- | --- | --- |
|  | **Basal** | **ZnO** | **BA+P** | **ZnO+BA+P** | **Basal** | **ZnO** | **BA+P** | **ZnO+BA+P** |
| Corn grain | 30.000 | 30.000 | 30.000 | 30.000 | 45.000 | 45.000 | 45.000 | 45.000 |
| Soybean meal | 10.000 | 10.000 | 10.000 | 10.000 | 15.000 | 15.000 | 15.000 | 15.000 |
| Whey powder | 22.895 | 22.895 | 22.895 | 22.895 | 16.870 | 16.870 | 16.870 | 16.870 |
| Corn Flour | 16.191 | 16.191 | 16.191 | 16.191 | 4.267 | 4.267 | 4.267 | 4.267 |
| Micronized Soy | 9.569 | 9.569 | 9.569 | 9.569 | 10.557 | 10.557 | 10.557 | 10.557 |
| Pig Plasma | 5.000 | 5.000 | 5.000 | 5.000 | 2.500 | 2.500 | 2.500 | 2.500 |
| Sugar | 2.500 | 2.500 | 2.500 | 2.500 | 2.000 | 2.000 | 2.000 | 2.000 |
| Calcium Sulphate | 0.550 | 0.550 | 0.550 | 0.550 | 0.000 | 0.000 | 0.000 | 0.000 |
| Dicalcium Phosp.20% | 0.489 | 0.489 | 0.489 | 0.489 | 0.965 | 0.965 | 0.965 | 0.965 |
| Zinc Oxid 72% |  | 0.350 |  | 0.350 |  | 0.350 |  | 0.350 |
| PX VEVOGUT P 25L^a^ |  |  | 0.400 | 0.400 |  |  | 0.400 | 0.400 |
| DL-Methionine | 0.196 | 0.196 | 0.196 | 0.196 | 0.198 | 0.198 | 0.198 | 0.198 |
| L-Tryptophan | 0.051 | 0.051 | 0.051 | 0.051 | 0.049 | 0.049 | 0.049 | 0.049 |
| L-Threonine | 0.103 | 0.103 | 0.103 | 0.103 | 0.120 | 0.120 | 0.120 | 0.120 |
| L-Lysine HCl | 0.288 | 0.288 | 0.288 | 0.288 | 0.306 | 0.306 | 0.306 | 0.306 |
| Ronozyme HiPhos GT^b^ | 0.010 | 0.010 | 0.010 | 0.010 | 0.010 | 0.010 | 0.010 | 0.010 |
| Arome | 0.075 | 0.075 | 0.075 | 0.075 | 0.075 | 0.075 | 0.075 | 0.075 |
| Antioxidant | 0.010 | 0.010 | 0.010 | 0.010 | 0.010 | 0.010 | 0.010 | 0.010 |
| Edulcorant | 0.055 | 0.055 | 0.055 | 0.055 | 0.055 | 0.055 | 0.055 | 0.055 |
| Salt | 0.250 | 0.250 | 0.250 | 0.250 | 0.250 | 0.250 | 0.250 | 0.250 |
| Vitaminic Premix^c^ | 0.050 | 0.050 | 0.050 | 0.050 | 0.050 | 0.050 | 0.050 | 0.050 |
| Mineral Premix^d^ | 0.200 | 0.200 | 0.200 | 0.200 | 0.200 | 0.200 | 0.200 | 0.200 |
| Kaolin | 1.519 | 1.169 | 1.119 | 0.768 | 1.518 | 1.168 | 1.118 | 0.768 |
| **Nutrients** |  |  |  |  |  |  |  |  |
| Metabolizable Energy, kcal | 3.561 | 3.561 | 3.561 | 3.561 | 3458 | 3458 | 3458 | 3458 |
| Crude protein, % | 20.593 | 20.593 | 20.593 | 20.593 | 21.473 | 21.473 | 21.473 | 21.473 |
| Digestible Lysine, % | 1.400 | 1.400 | 1.400 | 1.400 | 1.400 | 1.400 | 1.400 | 1.400 |
| Digestible Methionine, % | 0.466 | 0.466 | 0.466 | 0.466 | 0.480 | 0.480 | 0.480 | 0.480 |
| Digestible Met+Cystein, % | 0.826 | 0.826 | 0.826 | 0.826 | 0.826 | 0.826 | 0.826 | 0.826 |
| Digestible Threonine, % | 0.910 | 0.910 | 0.910 | 0.910 | 0.910 | 0.910 | 0.910 | 0.910 |
| Digestible Tryptophan, % | 0.280 | 0.280 | 0.280 | 0.280 | 0.280 | 0.280 | 0.280 | 0.280 |
| Digestible Valine, % | 0.966 | 0.966 | 0.966 | 0.966 | 0.966 | 0.966 | 0.966 | 0.966 |
| Lactose, % | 13.500 | 13.500 | 13.500 | 13.500 | 10.410 | 10.410 | 10.410 | 10.410 |
| Calcium, % | 0.625 | 0.625 | 0.625 | 0.625 | 0.625 | 0.625 | 0.625 | 0.625 |
| Total phosphorus, % | 0.487 | 0.487 | 0.487 | 0.487 | 0.582 | 0.582 | 0.582 | 0.582 |
| Available phosphorus, % | 0.482 | 0.482 | 0.482 | 0.482 | 0.548 | 0.548 | 0.548 | 0.548 |

^a^Commercial blend of benzoic acid and probiotic (Bacillus licheniformis, Bacillus subtilis and Enterococcus faecium NCIMB 10415); ^b^Commercial phytase with 10.000 FYT/g; ^c^Levels per kg of product: vitamin A (min) 6.000 IU; vitamin D3 (min) 1.500 IU; vitamin E (min) 15.000 mg; vitamin K3 (min) 1.500 mg; vitamin B1 (min) 1.350 mg; vitamin B2 4.000 mg; vitamin B6 2.000 mg; vitamin B12 (min) 20 mg; niacin (min) 20.000 mg; pantothenic acid (min) 9.350 mg; folic acid (min) 600 mg; biotin (min) 80 mg; selenium (min) 300 mg; ^d^Levels per kg of product: iron (min) 100 mg; copper (min) 10 mg; manganese (min) 40 g; cobalt (min) 1.000 mg; zinc (min) 100 mg; iodine (min) 1.500 mg.

**Supplementary Table 2.** Ingredients and calculated composition as-fed of the experimental diets (Starter phases I and II)

| **Ingredients** | **Starter I phase** | | | | **Starter II phase** | | | |
| --- | --- | --- | --- | --- | --- | --- | --- | --- |
|  | **Basal** | **ZnO** | **BA+P** | **ZnO+BA+P** | **Basal** | **ZnO** | **BA+P** | **ZnO+BA+P** |
| Corn grain | 50.000 | 50.000 | 50.000 | 50.000 | 65.000 | 65.000 | 65.000 | 65.000 |
| Soybean meal | 25.000 | 25.000 | 25.000 | 25.000 | 30.000 | 30.000 | 30.000 | 30.000 |
| Whey powder | 9.500 | 9.500 | 9.500 | 9.500 | 9.500 | 9.500 | 9.500 | 9.500 |
| Corn Flour | 5.000 | 5.000 | 5.000 | 5.000 | 5.000 | 5.000 | 5.000 | 5.000 |
| Micronized Soy | 3.082 | 3.082 | 3.082 | 3.082 | 0.000 | 0.000 | 0.000 | 0.000 |
| Sugar | 2.500 | 2.500 | 2.500 | 2.500 | 2.500 | 2.500 | 2.500 | 2.500 |
| Calcium Sulphate | 0.507 | 0.507 | 0.507 | 0.507 | 0.507 | 0.507 | 0.507 | 0.507 |
| Dicalcium Phosp.20% | 1.000 | 1.000 | 1.000 | 1.000 | 1.000 | 1.000 | 1.000 | 1.000 |
| Zinc Oxid 72% |  | 0.350 |  | 0.350 |  | 0.350 |  | 0.350 |
| PX VEVOGUT P 25L^a^ |  |  | 0.400 | 0.400 |  |  | 0.400 | 0.400 |
| L-Valine 96,5 | 0.127 | 0.127 | 0.127 | 0.127 | 0.122 | 0.122 | 0.122 | 0.122 |
| DL-Methionine | 0.247 | 0.247 | 0.247 | 0.247 | 0.228 | 0.228 | 0.228 | 0.228 |
| L-Tryptophane | 0.068 | 0.068 | 0.068 | 0.068 | 0.063 | 0.063 | 0.063 | 0.063 |
| L-Threonina | 0.234 | 0.234 | 0.234 | 0.234 | 0.238 | 0.238 | 0.238 | 0.238 |
| L-Lysine HCl | 0.523 | 0.523 | 0.523 | 0.523 | 0.543 | 0.543 | 0.543 | 0.543 |
| Arome | 0.075 | 0.075 | 0.075 | 0.075 | 0.075 | 0.075 | 0.075 | 0.075 |
| Ronozyme HiPhos GT^b^ | 0.010 | 0.010 | 0.010 | 0.010 | 0.010 | 0.010 | 0.010 | 0.010 |
| Antioxidant | 0.010 | 0.010 | 0.010 | 0.010 | 0.010 | 0.010 | 0.010 | 0.010 |
| Edulcorant | 0.050 | 0.050 | 0.050 | 0.050 | 0.000 | 0.000 | 0.000 | 0.000 |
| Salt | 0.300 | 0.300 | 0.300 | 0.300 | 0.300 | 0.300 | 0.300 | 0.300 |
| Vitaminic Premix^c^ | 0.050 | 0.050 | 0.050 | 0.050 | 0.050 | 0.050 | 0.050 | 0.050 |
| Mineral Premix^d^ | 0.200 | 0.200 | 0.200 | 0.200 | 0.200 | 0.200 | 0.200 | 0.200 |
| Kaolin | 1.518 | 1.168 | 1.118 | 0.768 | 1.516 | 1.166 | 1.116 | 0.766 |
| Nutrients |  |  |  |  |  |  |  |  |
| Metabolizable Energy, kcal | 3.387 | 3.387 | 3.387 | 3.387 | 3.246 | 3.246 | 3.246 | 3.246 |
| Crude protein, % | 19.529 | 19.529 | 19.529 | 19.529 | 19.794 | 19.794 | 19.794 | 19.794 |
| Digestible Lysine, % | 1.320 | 1.320 | 1.320 | 1.320 | 1.300 | 1.300 | 1.300 | 1.300 |
| Digestible Methionine, % | 0.495 | 0.495 | 0.495 | 0.495 | 0.478 | 0.478 | 0.478 | 0.478 |
| Digestible Met+Cystein, % | 0.779 | 0.779 | 0.779 | 0.779 | 0.767 | 0.767 | 0.767 | 0.767 |
| Digestible Threonine, % | 0.858 | 0.858 | 0.858 | 0.858 | 0.845 | 0.845 | 0.845 | 0.845 |
| Digestible Tryptophan, % | 0.264 | 0.264 | 0.264 | 0.264 | 0.260 | 0.260 | 0.260 | 0.260 |
| Digestible Valine, % | 0.911 | 0.911 | 0.911 | 0.911 | 0.897 | 0.897 | 0.897 | 0.897 |
| Lactose, % | 5.210 | 5.210 | 5.210 | 5.210 | 0.000 | 0.000 | 0.000 | 0.000 |
| Calcium, % | 0.625 | 0.625 | 0.625 | 0.625 | 0.749 | 0.749 | 0.749 | 0.749 |
| Total phosphorus, % | 0.533 | 0.533 | 0.533 | 0.533 | 0.436 | 0.436 | 0.436 | 0.436 |
| Available phosphorus, % | 0.494 | 0.494 | 0.494 | 0.494 | 0.380 | 0.380 | 0.380 | 0.380 |

^a^Commercial blend of benzoic acid and probiotic (Bacillus licheniformis, Bacillus subtilis and Enterococcus faecium NCIMB 10415); ^b^Commercial phytase with 10.000 FYT/g; ^c^Levels per kg of product: vitamin A (min) 6.000 IU; vitamin D3 (min) 1.500 IU; vitamin E (min) 15.000 mg; vitamin K3 (min) 1.500 mg; vitamin B1 (min) 1.350 mg; vitamin B2 4.000 mg; vitamin B6 2.000 mg; vitamin B12 (min) 20 mg; niacin (min) 20.000 mg; pantothenic acid (min) 9.350 mg; folic acid (min) 600 mg; biotin (min) 80 mg; selenium (min) 300 mg; ^d^Levels per kg of product: iron (min) 100 mg; copper (min) 10 mg; manganese (min) 40 g; cobalt (min) 1.000 mg; zinc (min) 100 mg; iodine (min) 1.500 mg.
